# Supplementary material for: Development of a Rapid Epstein–Barr Virus Detection System Based on Recombinase Polymerase Amplification and a Lateral Flow Assay
Source: Viruses. 2024 Jan 11;16(1):106. doi: 10.3390/v16010106 (PMC10818573; doi:10.3390/v16010106)
Supplement: Supplementary file 1 [file viruses-16-00106-s001.zip › viruses-2786811-supplementary.pdf]

Supplementary Material

Table S1-1 Cell lines used for experiments

Number	Cell line	Number	Cell line
1	104C1	26	BEAS-2B
2	143B/TK-	27	BeWo
3	293	28	BHK21
4	293T	29	BS-C-1
5	3T3-L1	30	BT
6	4T1	31	BV-173
7	769-P	32	C-33 A
8	786-O	33	CA46
9	A-204	34	Caco-2
10	A-431	35	Caov-3
11	A549	36	CCRF-CEM
12	A875	37	CCRF-SB
13	A9	38	CFPAC-1
14	ACHN	39	CHO/dhFr-
15	Aedes albopictus clone C6/36	40	CHO-K1
16	AN3 CA	41	CHSE-214
17	Anglne	42	CIK
18	ARH-77	43	COC1
19	B16-F0	44	COC1/DDP
20	B16-F1	45	CRFK
21	B6YH4	46	Daudi
22	B82	47	DLD-1
23	B95-8	48	DMS 153
24	BALB/3T3 clone A31	49	EAC
25	BC-3	50	EL-4-B5

Table S1-2 Cell lines used for experiments

Number	Cell line	Number	Cell line
51	EPC	76	Hep-G2/2.2.15
52	ES-2	77	HIC
53	Farage	78	Hi-five(BTI-Tn-5B1-4)
54	FG	79	HK-2
55	FRhk-4	80	HL-60
56	GIC	81	HOS
57	H22	82	Hs 578T
58	H22-H8D8	83	Hs 746T
59	H4	84	Hs68
60	H-4-II-E	85	HT-29
61	H9	86	HTR-8
62	H9C2	87	Huh-7
63	HaCaT	88	IBRS-2
64	HBZY-1	89	IMR-32
65	HCT 116	90	J774A.1
66	HCT-15	91	J82
67	HCT-8	92	Jurkat,cloneE6-1
68	HEC-1-B	93	JVM-2
69	HEL	94	K-562
70	HeLa	95	KATO III
71	HeLa 229	96	KG-1a
72	HeLa S3	97	KHOS-240S
73	Hep 3B2.1-7	98	KLE
74	Hep G2	99	KMB-17
75	Hepa 1-6	100	L1210

Table S1-3 Cell lines used for experiments

Number	Cell line	Number	Cell line
101	L8824	126	NCI-H292
102	L929	127	NCI-H929
103	LLC-MK2	128	NG108
104	LLC-PK1	129	NIH/3T3
105	LLC-WRC 256	130	NIH:OVCAR-3
106	L-M(TK-)	131	NK-92
107	LNCaP	132	NRK
108	LS 174T	133	OCI-AML3
109	M2-10B4	134	P3/NSI/1-Ag4-1[NS-1]
110	MA-104	135	P3X63Ag8.653
111	MA782/5s-8101	136	P815
112	MADB106	137	PA317
113	MC/CAR	138	PANC-1
114	MC3T3-E1	139	PC-12
115	MCF-7	140	PC-3
116	MDA-MB-435S	141	PCK
117	MDBK	142	PG-4(S+L-)
118	MDCK(NBL-2)	143	PK-15
119	MG-63	144	PT67
120	MGC803-1	145	Raji
121	MIA PaCa-2	146	RD
122	MKN45	147	RH-30
123	MOLT-4	148	RIN-m5F
124	MRC-5	149	RK13
125	MV3	150	RWPE-2

Table S1-4 Cell lines used for experiments

Number	Cell line	Number	Cell line
151	S-180	176	T24
152	S180-S2D9	177	T-47D
153	Saos-2	178	Tb 1 Lu
154	SH-SY5Y	179	TF-1
155	SiHa	180	TM4
156	SJSA-1	181	TT
157	SK-6	182	U14-GFP
158	SK-BR-3	183	U-2 OS
159	SK-HEP-1	184	U-251MG
160	SK-MES-1	185	U266B1
161	SK-N-SH	186	U-87MG
162	SK-OV-3	187	U-937
163	Sp2/0-Ag14	188	Vero
164	ST	189	Vero E6
165	STO	190	WEHI 164
166	SU-DHL-10	191	WI-38 VA-13 subline 2RA
167	SU-DHL-6	192	YAC-1
168	sVEC4-10		
169	SVP		
170	SW 1990		
171	SW 626		
172	SW1116		
173	SW1353		
174	SW480		
175	SYF		

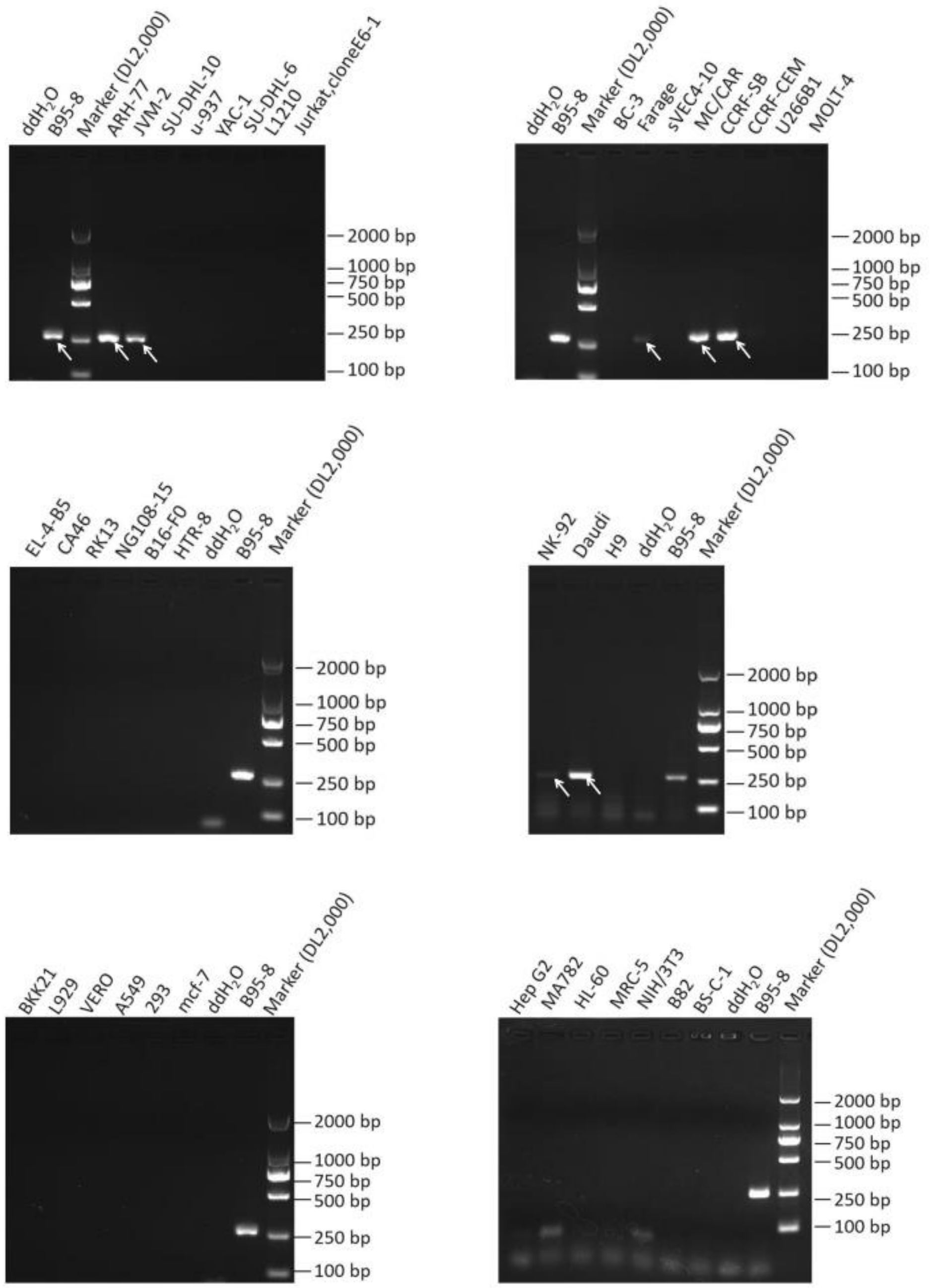

Figure S1-1 PCR system detection EBV agarose gel electropherogram

Positive cell lines: B95-8、ARH-77、JVM-2、Farage、MC/CAR、CCRF-SB、NK-92、Daudi

PS: B95-8 appears multiple times as a positive control, indicated only by an arrow once.

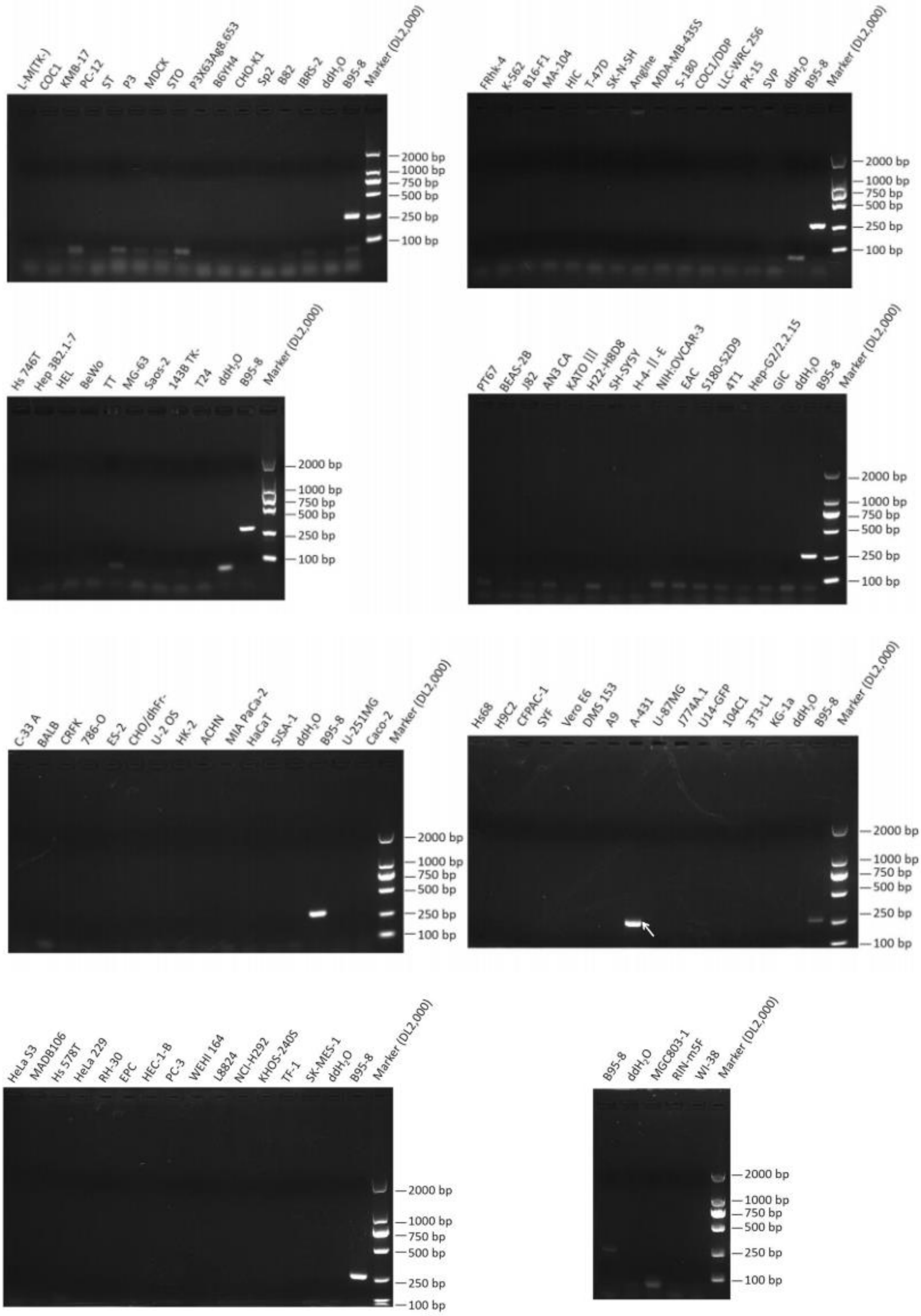

Figure S1-2 PCR system detection EBV agarose gel electropherogram

Positive cell lines: A-431

PS: B95-8 appears multiple times as a positive control, indicated only by an arrow once.

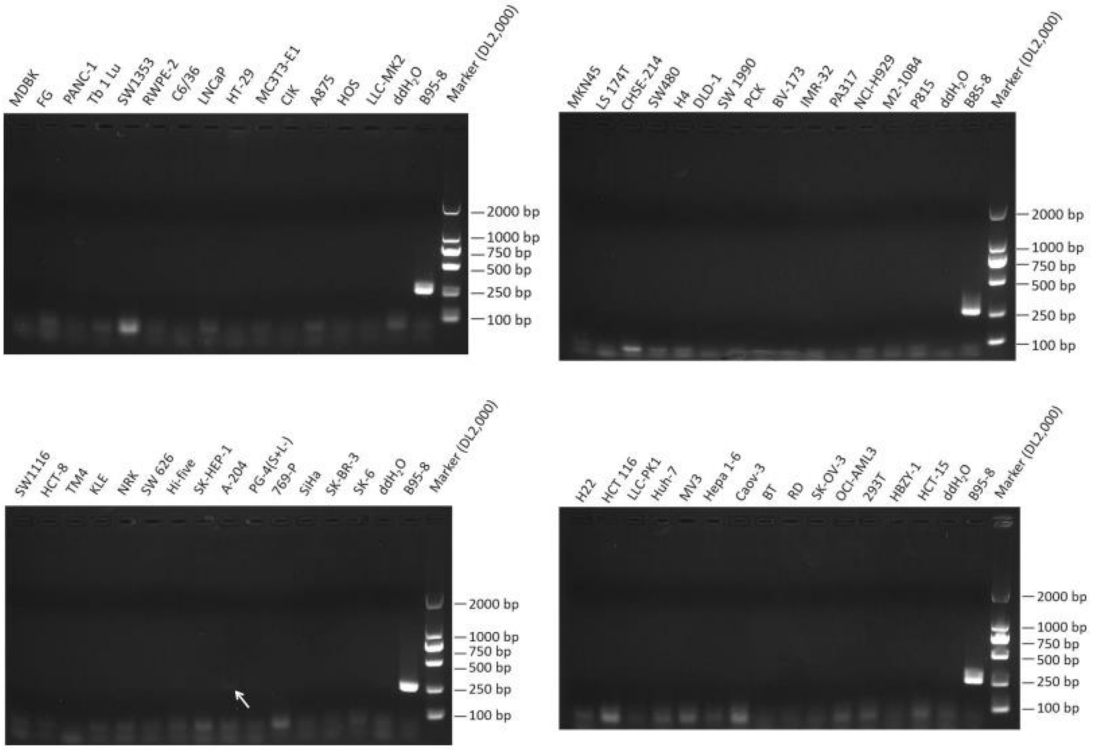

Figure S1-3 PCR system detection EBV agarose gel electropherogram

Positive cell lines: A-204

PS: B95-8 appears multiple times as a positive control, indicated only by an arrow once.

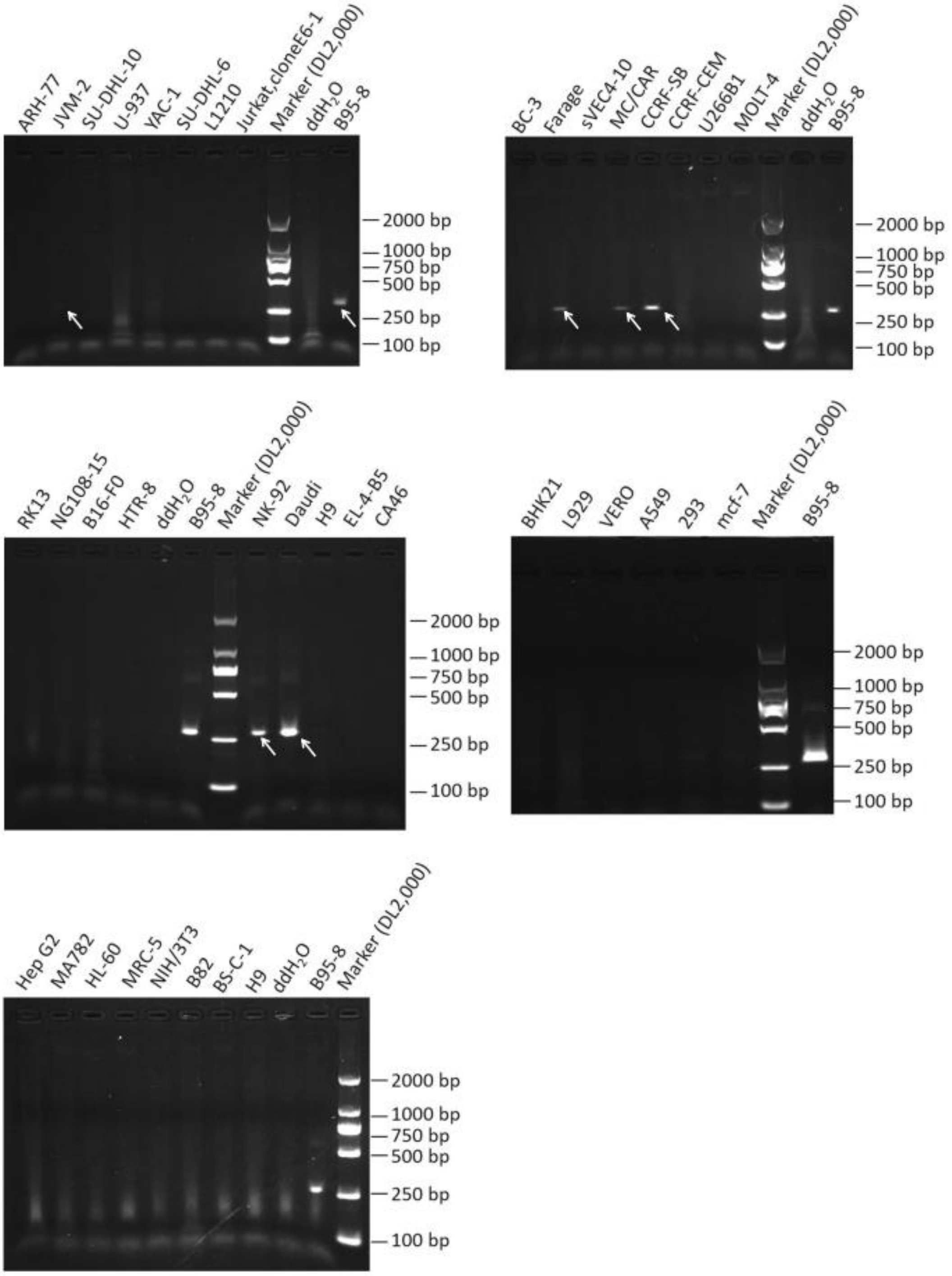

Figure 2-1 RPA system detection EBV agarose gel electropherogram

Positive cell lines: JVM-2、B95-8、Farage、MC/CAR、CCRF-SB、NK-92、Daudi

PS: B95-8 appears multiple times as a positive control, indicated only by an arrow once.

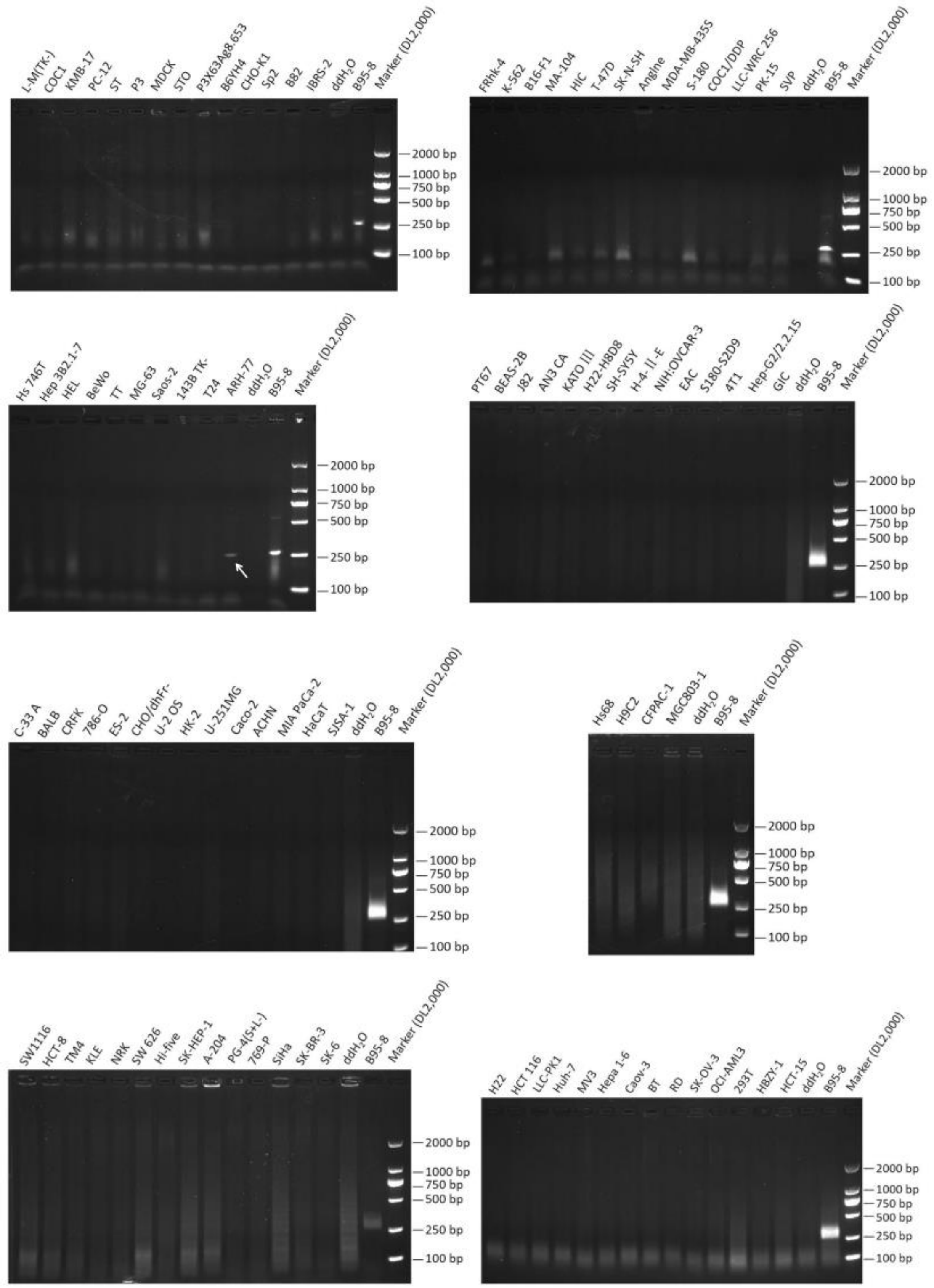

Figure S2-2 RPA system detection EBV agarose gel electropherogram

Positive cell lines: ARH-77

PS: B95-8 appears multiple times as a positive control, indicated only by an arrow once.

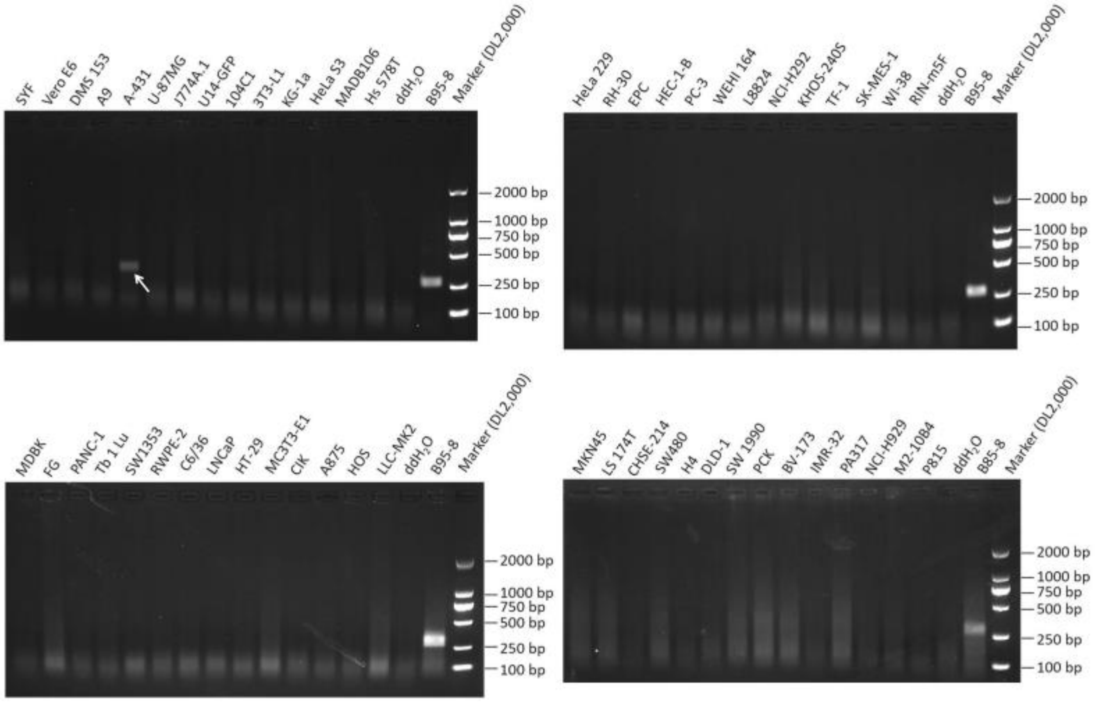

Figure S2-3 RPA system detection EBV agarose gel electropherogram

Positive cell lines: A-431

PS: B95-8 appears multiple times as a positive control, indicated only by an arrow once.

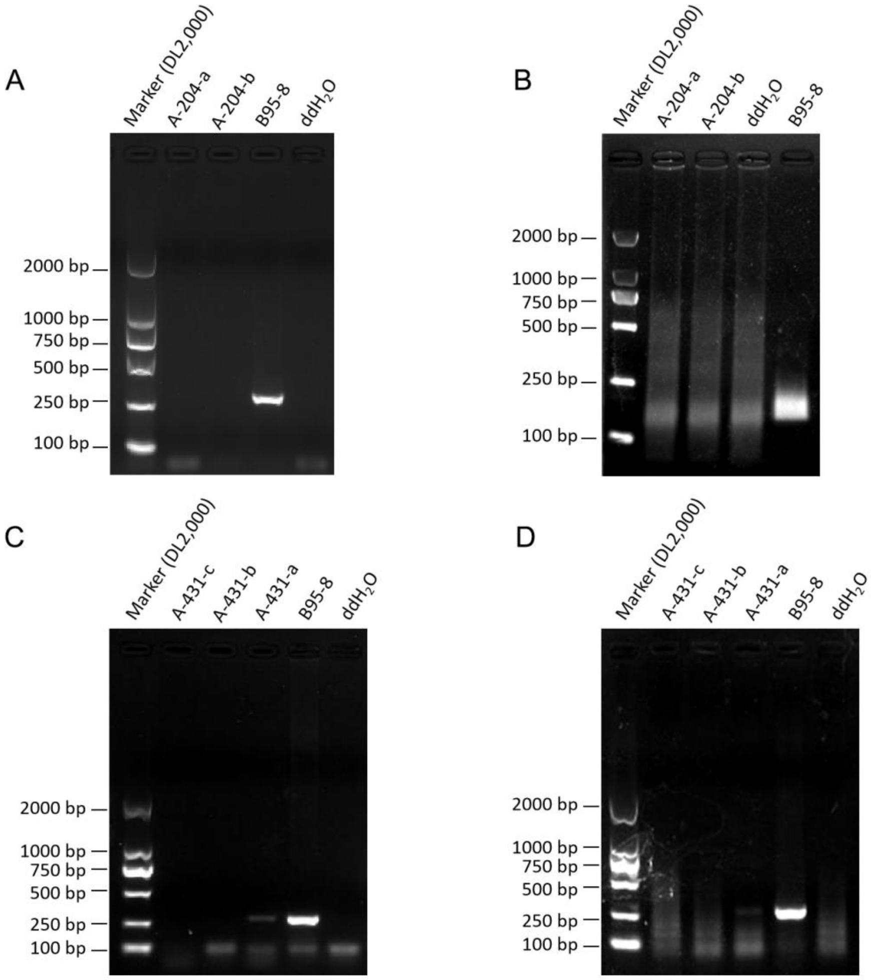

Figure S3 Detection of EBV in A-204 & A-431 cells by agarose gel electrophoresis

(A) PCR detection of EBV in different batches of A-204. (B) RPA detection of EBV in different batches of A-204. (C) PCR detection of EBV in different batches of A-431. (D) RPA detection of EBV in different batches of A-431.

PS: a. b and c respectively represent different batches

Sequencing Results of EBV Standard Plasmid Fragment

5’-CGGTCTGCGACAGATCGGATTACCGGTCCATGCACGTGTCCGACTACGG
CTATAACGAAGCAA-3’
